# Evaluation of a large-scale *E*-learning program on emotional regulation Tipi® for hospital workers and teachers in France during the COVID−19 pandemic

**DOI:** 10.1016/j.pmedr.2025.103208

**Published:** 2025-08-16

**Authors:** Xavier de la Tribonnière, Patrick Favennec, Marie Christine Picot, Valérie Macioce, Safa Aouinti, Pascale Clément, Marie Pommier, Luc Nicon

**Affiliations:** a Transversal Patient Education Unit (UTEP), CHU Montpellier, Univ Montpellier, Postal address: UTEP, Hopital La Colombière, 39 Avenue Charles Flahault, 34295 Montpellier Cedex 5, France; b Covea (Mutual Insurance Group Company)- Garantie Mutuelle des Fonctionnaires (GMF) (Mutual Guarantee for Civil Servants)), France; c Clinical Research and Epidemiology Unit, CHU Montpellier, Univ Montpellier, Postal adress : UTEP, Hopital La Colombière, 39 Avenue Charles Flahault, 34295 Montpellier Cedex, France; d CIC, INSERM U1411, CHU Montpellier, University of Montpellier, Montpellier, Postal address : CIC - Hôpital Gui de Chauliac, 80 Avenue Augustin Fliche, 34295 Montpellier Cedex 5, France; e Anxiety and Burnout Unit, CHU de Montpellier, Univ Montpellier, Montpellier, Postal address : UTEP, Hopital La Colombière, 39 Avenue Charles Flahault, 34295 Montpellier Cedex 5, France; f Tipi® Association, Postal address : 4, rue Pagès, 34070, Montpellier, France

**Keywords:** Education, distance, E-learning, Emotional regulation, Health personal, Teachers, COVID−19

## Abstract

**Objective:**

The Tipi® method is a rapid emotional regulation technique, focusing on physical sensations associated with an emotion, allowing both the sensations and the emotion to subside naturally. This study aimed to evaluate the effectiveness of an e-learning program on the Tipi® method in reducing stress and negative emotions among healthcare professionals and teachers during the COVID−19 pandemic.

**Methods:**

This prospective observational study was conducted online in 2020 and 2021. The program was offered to 82,000 hospital workers and 84,000 teachers in France (members of a mutual insurance company). It consisted of two modules, with online assessments. The primary outcome was the change in stress level (on a 0–10 scale), from baseline to three months.

**Results:**

2648 individuals registered for the program, and 456 completed the assessment questionnaire. Follow-up rates were 47 % at one month and 31 % at three months. The first module was completed by 76 % and 19 % completed both. Stress and emotions significantly decreased at one month and three months, with a median reduction of one point on a 10-point scale, considered clinically meaningful at population level. Stress decreased by 14 % at one month (*p* < 0.01) and three months (p < 0.01). Anxiety reductions were 14 % at one month (p < 0.01) and 17 % at three months (p < 0.01). Greater improvements were observed in those who practiced the Tipi® method. Satisfaction was moderate (e-learning, 7/10. Tipi® method 6/10).

**Conclusions:**

The Tipi® e-learning program showed feasibility, acceptability, and effectiveness in reducing stress and negative emotions among hospital workers and teachers.

## Highlights

• The Tipi® method is a novel approach for rapid emotional regulation
• *E*-learning program on the Tipi® was offered to hospital workers and teachers
• E-learning showed safety and effectiveness for self-guided learning
• The Tipi® method may be of interest for rapid stress and emotion regulation

## 1 Introduction

The COVID−19 pandemic significantly heightened stress levels, impacting mental and overall health across the global population (Salari et al., 2020; Galea et al., 2020; Dubey et al., 2020; Pfefferbaum and North, 2020). Healthcare professionals and teachers, as front lines workers, were particularly vulnerable due to their ongoing exposure to the virus and job demands (Lai et al., 2020; Shanafelt et al., 2020; Li et al., 2021a; Bow et al., 2023; Silva et al., 2021). A meta-analysis identified frequent issues such as insomnia, burnout, phobias and anxiety disorders in these groups (Silva et al., 2021; Li et al., 2021b; Stachteas and Stachteas, 2020).

The situation underscored the urgent need for effective emotional regulation tools (Dai et al., 2021). Various methods have emerged, including cognitive-behavioral therapy (CBT), mindfulness, Acceptance and Commitment Therapy (ACT), expressive writing, relaxation, cardiac coherence, and Eye Movement Desensitization and Reprocessing (EMDR). These techniques have demonstrated stress and anxiety reduction between 20 % to 38 % (Khoury et al., 2015; Goyal et al., 2014; Gloster et al., 2020), with meta-analyses supporting their role in preventing burnout among healthcare workers and teachers (Heber et al., 2017; Harrer et al., 2019). The widespread use of such strategies also improved physical health and well-being (Dai et al., 2021), while digital platforms proved beneficial for motivation and learning (Noor et al., 2022).

However, many techniques require long-term practice or therapeutic guidance, limiting their use in high-pressure environments. Thus, a need remains for accessible, rapid and self-directed tool. The Tipi® method offers a somatic, non-cognitive approach. Developed by Luc Nicon in the early 2000s and refined over time, it was based on pedagogical research into emotional blocks in learning (Nicon, 2003). A study from 2003 and 2006 involving 350 individuals with various psychological challenges (e.g., fear, phobias, sadness, anger, inhibition, depression) reported a 90 % to 99 % success depending on emotion type (Nicon, 2019a; Nicon, 2019b). Another case study describes significant weight loss in overweight individuals following regulation of food-related emotions (Bertin et al., 2021). The Tipi® method has gained international recognition, with several books published and translated into multiple languages (Nicon, 2003; Nicon, 2007; Nicon, 2013).

To broaden access, the mutual insurance group company Covea, supported an e-learning adaptation.

Based on promising results observed in-person training (Favennec and De la Tribonnière, 2021), the program offered free to healthcare workers and teachers insured by Garantie Mutuelle des Fonctionnaires (Mutual Guarantee for Civil Servants) (GMF), a member of Covea.

We hypothesized that, during this highly stressful period of the pandemic, the Tipi® method could help individuals reduce their stress by learning and applying the technique autonomously. The aim of our study was to assess the impact of the Tipi® e-learning program on stress and emotion regulation among hospital workers and teachers in France during 2020 and 2021. To better understand the impact of the intervention, we also examined whether its effectiveness varied across different subgroups (age, gender, occupation) and according to the practice of the method or the number of successful regulations.

## 2 Methods

### 2.1 Description of the study

We conducted a prospective observational study to assess the acceptability, feasibility, and effectiveness of a Tipi® e-learning program among hospital workers and teachers insured by the GMF. The local ethics committee of Montpellier University Hospital approved the study in May 2020 (No. 198711).

An invitation email was sent to eligible professionals. At the start of the e-learning course, participants received written and video information and gave digital consent to participate.

Participants were adult GMF members enrolled in the program who voluntarily agreed to take part in this study. There were no exclusion criteria.

Data storage complied with the General Data Protection Regulation in Europe.

### 2.2 Description of the Tipi® emotional regulation method

The Tipi® method has two goals: alleviate disruptive emotions and enabling users to apply it autonomously.

It involves focusing on physical sensations linked to an emotional experience, either in real time or memory recall. These sensations are observed without judgment or control, allowing them to evolve naturally for one to two minutes. They may change or disappear completely. Some situation may require two or three repetitions for complete resolution (Nicon, 2007; Nicon, 2013).The Tipi® method differs from other techniques by its speed and lasting relief. Although training with a certified practitioner is generally recommended, some individuals can learn it through self-practice, especially with well-designed e-learning tool.

### 2.3 *E*-learning program description

The e-learning course was developed by a multidisciplinary team and tested before deployment. It includes two modules with eight missions in Module 1 and six in Module 2.

• Module 1 introduces the Tipi® method. It includes an information video, testimonials, two motion designed videos on physiological stress response and how Tipi® method works, and two practical quiz practice exercises.
• Module 2 deepens the learning. It includes a motion design video on common pitfalls (e.g., focusing too much on a physical sensation or intellectualizing the process), and explores extended uses of Tipi®. Optional support from certified practitioners was available

Each mission lasts a few minutes and must be completed in sequence. Participants can revisit previous missions completed steps but cannot skip forward. Additional resources include downloadable PDFs, a “Frequently Asked Questions” section, and live chat with certified professionals. Total estimated training time is estimated at one to two hours.

Data Collection and Quality Control.

Date were collected via secure online questionnaires by a third-party provider. All data were anonymized and compiled on that platform, then deleted after being transferred to the University Hospital of Montpellier in compliance with General Data Protection Regulation in Europe.

Enrollment statistics, including the number of participants completing each module, were recorded for the scientific study.

### 2.4 Description of evaluation questionnaires

A baseline questionnaire with seven questions gathered demographic data (e.g., gender, age group, profession), prior experience with the Tipi® method, and self-assessed stress and emotion levels (fear, anxiety, sadness, anger) on a scale from zero to 10. Fear and anxiety were distinguished: fear refers to immediate responses to identifiable dangers, while anxiety describes prolonged responses to anticipated threats.

A follow-up questionnaire, emailed one month later, included 14 questions to reassess stress and emotions on a zero to 10 scale. Participants were asked whether they had used the Tipi® method, the frequency of its practice, and its perceived success (i.e. when the regulated stress or emotion disappeared). They were also asked their opinion on the importance of being able to use this method independently (4 possible choices), and whether they would recommend the e-learning program to a colleague.

Difficulties, reasons for non-use (e.g., no opportunity, lack of confidence) and any adverse effects were also collected. Satisfaction with the Tipi® method and e-learning program was rated separately on a zero to 10 scale.

The same questionnaire was emailed three months later.

Details of these questionnaires are provided in the supplementary data.

### 2.5 Outcomes

The primary outcome was the absolute variation in self-reported stress levels between baseline and three months on a zero to 10 scale. Secondary outcomes included changes in stress and emotions (fear, anxiety, sadness, anger) at one month and three months, completion rates for each e-learning module, successful applications of the Tipi® method, and participants satisfaction.

### 2.6 Methodology and statistics

Participants characteristics and evaluation criteria were summarized for the total study population and separately for hospital workers and teachers. Quantitative variables were described using medians and interquartile ranges, while categorical variables were presented as frequencies and percentages.

Comparisons between groups used the Chi-squared test or Fisher's exact test for categorical variables and the Student's *t*-test or Wilcoxon Mann-Whitney test for quantitative variables, depending on distribution normality.

Stress and emotion levels were assessed at baseline, one and three months, in the total study population and within specific groups (teachers and hospital workers). Absolute variations as well as relative variations (expressed as a percentage of the baseline score) between baseline and one month and between baseline and three months, were calculated and expressed with the median (interquartile range (IQR)). All variations were analyzed using paired Student's *t*-tests or Wilcoxon tests if normality conditions were not met. Subgroup analyses were also carried out to compare stress and emotion levels at each timepoint and their variation according to gender, age (≤35y vs 36–50 vs > 50y), group (teachers vs hospital workers), and occupation within each group (for teachers: administrative staff and others vs primary school teacher vs secondary school/high school/higher education teachers; and for hospital workers: administrative staff and others vs doctors/pharmacists vs paramedics). Mann-Whitney test, Kruskal-Wallis test or ANOVA were used as appropriate.

Absolute variations in stress and emotion levels between baseline and one month for the total study population were also assessed and compared depending on the practice of the method and the number of successful regulations.

Statistical analyses were conducted using SAS® 9.04 (SAS Institute Inc., Cary, NC, USA) and figures were generated with R 4.5.0.

## 3 Results

### 3.1 Description of the study population

**Fig. 1:**
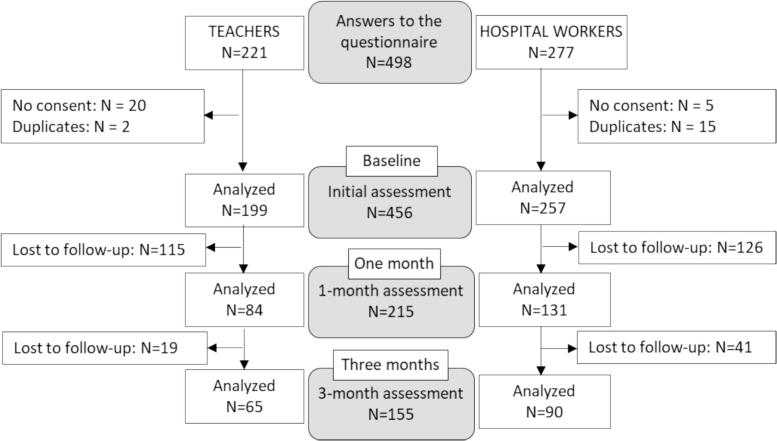
Flow chart of the e-learning evaluation study on the Tipi® emotional regulation method in hospital workers and teachers (France, 2020–2021).

A total of 166,000 emails were sent to GMF members in the education and healthcare sectors. Hospital workers received 82,000 emails in two waves (October 2020 and January 2021), while 84,000 emails were sent to teachers in March 2021. The email opening rate was 25 %, and 2648 individuals registered for the e-learning program (1.6 % of all recipients or 6.4 % of those who opened the email). Among these, 498 participants agreed to take part in the scientific evaluation study. Ultimately, 456 participants (199 hospital workers and 257 teachers) signed informed consent forms and completed the first questionnaire. However, participation declined over time, with 47 % completing the evaluation questionnaire at one month and 31 % at three months (Fig. 1).

Baseline characteristics were similar between hospital workers and teachers, except for age distribution: a higher proportion of teachers were aged 36 to 50, while a greater number of hospital workers were aged 25 to 35 (Table 1). Most participants (97 %) had no prior experience with the Tipi® emotional regulation method. A wide variety of professions within the hospital and education sectors were represented. Baseline stress and emotional levels were comparable between the two groups.

**Table 1:** Basic sociodemographic characteristics of the overall French study population, as well as in the teacher and hospital workers subgroups enrolled in the Tipi® emotional regulation method (France, 2020–2021).

Variables	Total population n (%)	Teachers n (%)		Hospital workers n (%)		P
	*n* = 456	*n* = 199		*n* = 257		
Sex, women	385 (84)	163 (82)		222 (86)		0.19^1^
Age						0.04^2^
< 25 years	6 (1)	4 (2)		2 (1)		
25–35 years	74 (16)	22 (11)		52 (20)		
36–50 years	219 (48)	103 (52)		116 (45)		
> 50 years	157 (35)	70 (35)		87 (34)		
Professional activity (working/retired)	446 (98) / 10 (2)	194 (98) / 5 (2)		252 (98) / 5 (2)		0.75^2^
Previous practice of the Tipi® method	13 (3)	4 (2)		9 (4)		0.34^1^
Occupation			N = 194^3^		N=251^3^	
		Primary school teacher	52 (27)	Doctors	18 (7)	
		Secondary school teacher	23 (12)	Nurse	92 (36)	
		High school teacher	35 (18)	Orderly	42 (17)	
		Higher education teacher	10 (5)	Paramedics	25 (10)	
		Head of School	10 (5)	Pharmacist and similar	8 (3)	
		Other educational staff⁎	18 (9)	Health executive	12 (5)	
		Medical and social staff	7 (4)	Administrative staff	28 (11)	
		Administrative staff	26 (13)	Technician	17 (7)	
		Laboratory technician	6 (3)	other⁎⁎⁎	9 (4)	
		other⁎⁎	7 (4)			

p-values are calculated using the Chi-square test or Fisher's exact test, depending on the validity conditions of the tests.

⁎ Other educational staff: librarian, educational advisor, support staff for students with disabilities, educational assistant.

⁎⁎ Other (teachers): business executive, student, trainer, scientific project manager, etc.

⁎⁎⁎ Other (hospital workers): sales executive, student, trainer, cleaning staff, etc.

1 Chi-squared test.

2 Fisher's exact test.

3 The number of participants who answered the occupation question.

### 3.2 *E*-learning take-up rate

Among the 456 participants included in the scientific study, 76 % completed the first e-learning module, which focuses on learning the Tipi® method and its independent application. Only 19 % completed both modules.

### 3.3 Emotional regulation

**Fig. 2:**
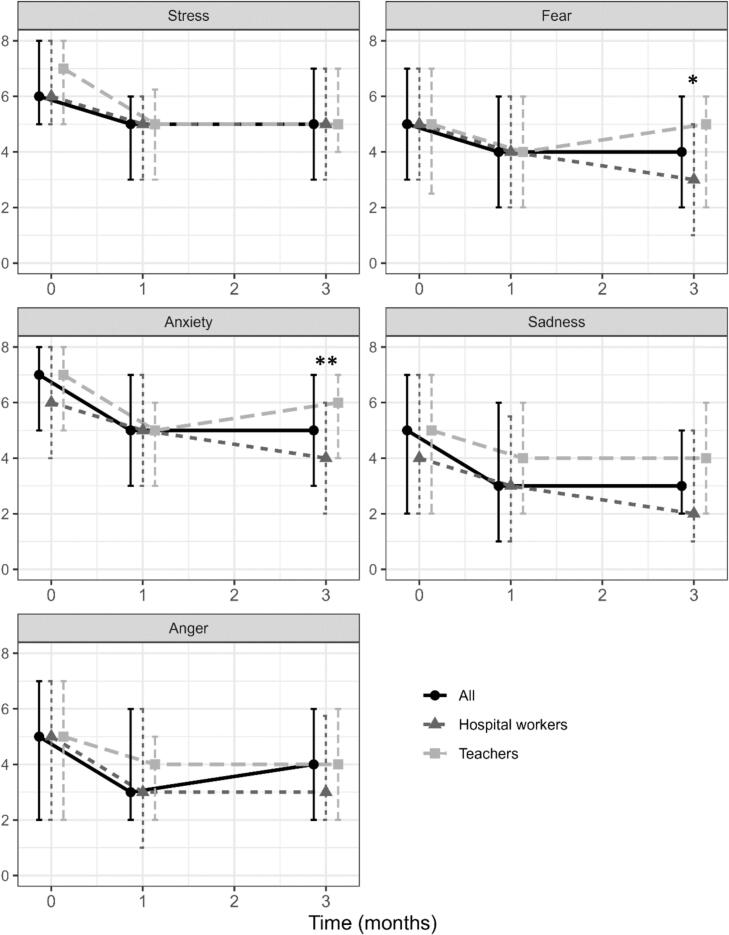
Median levels of stress and emotions at baseline, one month and three months the overall French study population, as well as in the teacher and hospital worker subgroups enrolled in the Tipi® emotional regulation method (France, 2020–2021). Scores range from zero (none) to 10 (maximum value). Bars represent the interquartile range. * and ** indicate statistically significant differences between groups at the indicated time point, with *p* < 0.05 and *p* < 0.01, respectively (Wilcoxon- Mann-Whitney test). ^1^ Stress and emotions (fear, anxiety, sadness, anger) were self-assessed using the TIPI® method, with scores ranging from 0 (none) to 10 (maximum).

Stress and all four measured emotions (fear, anxiety, sadness, anger) significantly decreased at one month, compared to baseline in the overall study population, as well as among hospital workers and teachers (Fig. 2, Supplementary Table 1). The only exception was fear in hospital workers, where the reduction was borderline significant (*p* = 0.06). The median reduction in visual analogue scale scores across all measures was 1 point. At three months, stress and all four emotions remained significantly reduced in the overall study population and among hospital workers as compared to baseline. Among teachers, only stress and anxiety remained significantly lower at three months than at baseline (Supplementary Table 1).

When considering relative reductions from baseline values, stress decreased by 14 % at both one month (*p* < 0.01) and three months (p < 0.01). Anxiety showed a 14 % reduction at one month (p < 0.01) and 17 % at three months (p < 0.01), with reductions reaching 24 % among hospital workers. Sadness decreased by 17 % at one month (*p* = 0.04), while anger decreased by 13 % at one month (p < 0.01) (Supplementary Table 2).

Subgroups analyses according to gender, age and occupation within each group are displayed in Supplementary figs. 1 to 4, respectively. Fear at baseline and stress at one month were higher in women than in men. No other difference was observed according to gender, age, or according to occupation among teachers. Among hospital workers, higher levels of sadness and anger were observed at one month in administrative staff. Absolute baseline to one month variations did not differ between subgroups, except for sadness and anger according to teacher occupation subgroups and fear according to gender, but none of the baseline-three months variations differed between subgroups.

Another subgroup analysis compared participants who reported at one month, not practicing the method (*n* = 105, 49 %) with those who reported practicing the method (*n* = 109, 51 %) and with those who succeeded in regulating more than one (*n* = 95, 44 %), more than three (*n* = 43, 20 %), or more than five regulations (*n* = 19, 9 %). Absolute and relative changes in scores showed greater reduction in anxiety, sadness, and anger among participants who practiced the Tipi® method and among those who successfully completed multiple emotion regulations, compared to those who did not use the method (Table 2).

**Table 2:** Absolute and relative changes in stress and emotion scores between baseline and one month (N=214^1^), according to the practice of the Tipi® method and the number of successful emotion regulations, in the overall French study population as well as in the teacher and hospital worker subgroups enrolled in the Tipi® emotional regulation method (France, 2020–2021).

	n	did not practice	practiced	p^4^	practiced and succeeded ≥1 times	p^4^	practiced and succeeded ≥3 times	p^4^	practiced and succeeded ≥5 times	p^4^
			n = 109		n = 95		n = 43		n = 19	

Absolute changes^2^ from baseline to one month										
stress^5^	102	-1 (−2; 1)	-1 (−2; 0)	0.5	-1(−2; 0)	0.4	-1 (−2; 1)	0.5	−2(−3; 1)	0.5
fear	105	0 (−2; 1)	−1 (−2; 1)	0.4	−1 (−2; 1)	0.2	−1 (−3; 1)	0.3	-2(−3; 1)	0.2
anxiety	105	0 (−2; 1)	−1 (−2; 0)	0.06	−1(−3; 0)	0.03	−1 (−3; 1)	0.1	-2(−4; −1)	0.01
sadness	105	0 (−2; 1)	−1 (−3; 0)	<0.01	−1(−3; 0)	<0.01	-1(−2; 0)	0.05	-1(−4; −1)	<0.01
anger	105	0 (−2; 1)	-1 (−3; 0)	0.03	-1(−4; 0)	0.02	-1(−4; 0)	0.03	-1(−4; 0)	0.06

Relative changes^3^ from baseline to one month										
stress^5^	102	−14 (−40; 20)	−14 (−33; 0)	0.8	−14 (−38; 0)	0.7	−14 (−33; 14)	0.8	−25 (−50; 33)	0.8
fear	105	0 (−40; 40)	−22 (−40; 33)	0.3	−25 (−43; 25)	0.2	−25 (−57; 100)	0.4	−29 (−57; 50)	0.2
anxiety	105	0 (−33; 14)	−20 (−43; 0)	0.08	−22 (−43; 0)	0.04	−22 (−50; 33)	0.1	−44 (−57; −11)	0.01
sadness	105	0 (−8; 40)	−25 (−63; 0)	<0.01	−29 (−63; 0)	<0.01	−20 (−50; 0)	0.1	−29 (−78; −17)	<0.01
anger	105	0 (−33; 17)	−25 (−56; 0)	0.04	−25 (−56; 0)	0.06	−25 (−57; 0)	0.07	−25 (−63; 0)	0.1

1 N = 214 due to loss to follow-up before the one-month evaluation. Among the 215 participants shown in the flowchart (Fig. 1), one participant had missing data regarding the use of the Tipi® method.

2 Absolute and relative changes are reported as the medians (interquartile ranges).

3 Relative changes in visual analogue scale scores are calculated as percentage of the baseline score.

4 *p*-values correspond to comparisons with the “did not practice” subgroup using the Wilcoxon-Mann-Whitney test.

5 For the stress item, 3 participants did not provide a response (*n* = 102 instead of 105).

Among participants who did not practice the method, the most common reasons were forgetting to use it (52 % at one month, 69 % at three months), lack of opportunity (28 % at one month, 14 % at three months), lack of desire (10 % at both one month and three months), and feeling incapable (10 % at one month, 6 % at three months).

### 3.4 Difficulties and satisfaction

At one month, 52 % of participants reported difficulties in practicing the Tipi® method, increasing to 58 % at three months. Common challenges included identifying physical sensations, allowing sensations to evolve, confirming the completion of regulation, and managing recurring or persistent emotional difficulties.

Adverse effects were reported by 6 % of participants at one month and 5 % at three months. These included difficulties in letting emotions evolve, fatigue, low mood, fear of failure, heightened sensitivity, and physical sensations linked to unresolved emotions or the regulation process itself.

Median satisfaction scores for the e-learning program were 7/10 (IQR: 5–8) at one month and 6/10 (IQR: 4–8) at three months. Satisfaction with the Tipi® method was slightly lower, with median scores of 6/10 (IQR: 4–7) at one month and 5/10 (IQR: 3–7) at three months. These results were consistent across subgroups.

Regarding the recommendation of the Tipi® method to colleagues or peers, 73 % of participants responded “definitely yes” or “probably yes” at one month, decreasing to 68 % at three months.

A large majority (95 %) of participants expressed a preference for autonomous emotional regulation, and nearly half (45 %–47 % at one month and three months) indicated an interest in professional training in the Tipi® method.

## 4 Discussion

This study highlighted the feasibility and preliminary effectiveness of learning the Tipi® emotional regulation method through a large-scale e-learning program among hospital workers and teachers during the COVID-19 pandemic. To our knowledge, this is one of the first large-scale evaluations of an emotional self-regulation technique delivered via digital learning and supported by a scientific assessment conducted under real-world conditions.

Indeed, very few e-learning programs for healthcare professionals and teachers have focused on stress reduction. Some programs have addressed cognitive reappraisal (García-Batista et al., 2021) or meditation (Salcido-Cibrián et al., 2019). One digital program for healthcare professionals, which included expert advice on mental well-being and frontline pandemic management, demonstrated stress reduction benefits (Blake et al., 2020).

The median reduction in stress was 1 point out of 10 at one month, maintained at three months, corresponding to a 14 % relative reduction from baseline to one month and three months, with anxiety reductions reaching up to 17 % in the whole study population and 24 % among hospital workers. These results are consistent with previous studies showing that digital interventions for stress and emotion regulation lead to relative improvements ranging from 10 % to 25 % (Heber et al., 2017; Harrer et al., 2019).

A relative reduction of 10 % or more in stress levels is generally considered clinically meaningful at the study population level, particularly in the context of large-scale public health or occupational health interventions (Dreison et al., 2018; Cohen and Janicki-Deverts, 2012). Even small variations in average stress scores can translate into substantial health and productivity benefits across large groups, by reducing the risk of chronic illnesses, burnout, absenteeism, and emotional exhaustion.

In this study, a positive association has been observed between the extent of stress or emotional reduction and the intensity of practice. This finding is consistent with a meta-analysis by Strohmaier (2020), which demonstrated a dose–response relationship between the intensity of meditation programs and improvements in depression, stress, and anxiety outcomes (Strohmaier, 2020).

Moreover, the 14 % reduction in stress observed at three months was achieved despite the fact that only 19 % of participants completed the entire e-learning program, which further supports the potential effectiveness of the Tipi® method.

One of the main strengths of this study lies in the scalability and minimal resources required: the free e-learning program completed in under two hours, was carefully designed and tested.

Unlike other emotional regulation techniques requiring long-term support (e.g., CBT, mindfulness, ACT), the Tipi® method uses brief somatic awareness for rapid resolution, suiting busy frontline professionals.

Strong participants' interest was noted: 95 % valued autonomous use and nearly half desired professional training.

Despite these promising results, this study also has limitations. The completion rate for the full e-learning program was low (19 %), similar to other massive open online course based interventions in the healthcare sector (Vaona et al., 2018; Regmi and Jones, 2020; Chi et al., 2023), due to time constraints, lack of motivation, insufficient commitment, forgetfulness, uncertainty about how to apply the method, or limited social support (Eriksson et al., 2016; Feng et al., 2017).

Maintaining motivation is a known challenge in e-learning, as shown in nursing (Alfaleh et al., 2023). Parallel support during the e-learning program might have helped sustain motivation or address challenging aspects, as suggested in another study (Liu et al., 2022). Several studies have shown that learning emotional regulation techniques via e-learning may be less effective than when guided by a trained professional. For example, in the case of sophrology or mindfulness-based programs, instructor-led sessions, whether in person or via videoconference, tend to result in better adherence, deeper emotional engagement, and more significant outcomes than purely self-directed formats (Khoury et al., 2015; Goyal et al., 2014; van Rangelrooij et al., 2020).

Comparison with a reference group who did not practice the Tipi® method revealed better reductions in stress and certain emotions among participants who did use the method. However, the observational study was initially designed without a control group due to the constraints and urgency of the pandemic, which limits causal inference. A placebo effect or contextual confounding factors cannot be ruled out. Randomized comparative studies, with other validated emotional regulation techniques are needed.

*E*-learning satisfaction was moderate (7/10), consistent with similar studies, possibly due to the lack of human support. For instance, two studies on e-learning during COVID-19 reported satisfaction rates ranging from 45 % to 58 % among medical students (Nakhoda et al., 2021; Güllü et al., 2022).

Tipi® satisfaction was also moderate (6/10). This may reflect difficulties in adopting the method in a self-directed e-learning context with a high dropout rate.

There are other emotional regulation methods, such as CBT, mindfulness-based stress reduction (MBSR), sophrology, emotional expression through writing or art, relaxation, cardiac coherence, EMDR, and physical exercise. Among them, several validated approaches involving in-person support have demonstrated their effectiveness in emotional regulation.

In the context of in-person learning, sophrology, although still not widely evaluated through large-scale randomized controlled trials, has shown average stress reductions of 20 % to 30 % in contexts such as chronic pain, maternity, or stressful work environments (van Rangelrooij et al., 2020). ACT has been shown to reduce stress and anxiety by 20 % to 35 %, particularly in patients with anxiety disorders or in high-pressure professional settings (Gloster et al., 2020). The MBSR program is one of the most well-documented: several meta-analyses report reductions in perceived stress ranging from 25 % to 38 % after 8 weeks, with effects maintained at 3 and 6 months (Khoury et al., 2015; Keng et al., 2011). Finally, mindfulness meditation, even outside the structured MBSR framework, has been associated with improvements in emotional well-being and stress reductions of 15 % to 30 %, depending on the duration and regularity of practice (Goyal et al., 2014; Hölzel et al., 2011). Regarding the Tipi® method, there are currently no established scientific date assessing stress reduction following guided in-person sessions as part of a multi-session program, but studies in this direction are currently being developed. This study adds to the available evidence on the Tipi® method that showed encouraging efficacy across a spectrum of emotions, which did not reappear in similar situations, even after one year (Nicon, 2019a; Nicon, 2019b).

Unlike traditional techniques that develop emotional skills progressively, the Tipi® method aims for a rapid and often permanent resolution of emotional disturbances. If validated, Tipi® method could complement or even replace conventional therapies for rapid autonomous emotional regulation.

## 5 Conclusion

Although only 19 % of participants completed the entire e-learning program, our findings demonstrate the feasibility, safety, and effectiveness of autonomous stress and emotion regulation using the Tipi® method, without professional support. These results highlight the value of both the e-learning program and the Tipi® method itself.

Given its accessibility and potential for large-scale dissemination, particularly through e-learning, Tipi® could represent a valuable complementary tool for healthcare professionals, educators, and individuals seeking a self-directed emotional regulation strategy. Although further evidence is needed, our results suggest that the Tipi® method could secure its place within the therapeutic landscape of emotion regulation methods. Its advantages include simplicity, speed, sustained effectiveness, ease of learning, and the potential for rapid autonomy.

To strengthen these preliminary findings, longer-term studies will be necessary to assess the stability of the emotional effects achieved through this method. In addition, its application should be explored in other contexts, such as the prevention of anxiety disorders, support for burnout, or among specific populations such as students, caregivers, or patients with chronic illnesses or chronic pain. Finally, the implementation of randomized controlled trials comparing Tipi® to a placebo or to other validated emotional regulation techniques would be a crucial step in confirming its effectiveness, clarifying its optimal indications, and guiding its integration into broader mental health strategies.

## Declaration of generative AI

During the preparation of this work the author(s) used ChatGPT04 in order to improve language and readability, with caution. After using this tool/service, the author(s) reviewed and edited the content as needed and take(s) full responsibility for the content of the publication.

## CRediT authorship contribution statement

**Xavier de la Tribonnière:** Writing – review & editing, Writing – original draft, Visualization, Validation, Supervision, Resources, Project administration, Methodology, Investigation, Conceptualization. **Patrick Favennec:** Writing – review & editing, Software, Project administration, Investigation, Funding acquisition, Data curation, Conceptualization. **Marie Christine Picot:** Writing – review & editing, Validation, Methodology, Investigation, Formal analysis, Data curation, Conceptualization. **Valérie Macioce:** Writing – review & editing, Writing – original draft, Validation, Formal analysis, Data curation. **Safa Aouinti:** Writing – review & editing, Formal analysis, Data curation. **Pascale Clément:** Writing – review & editing, Visualization, Validation, Methodology, Conceptualization. **Marie Pommier:** Writing – review & editing, Visualization, Validation, Methodology, Conceptualization. **Luc Nicon:** Writing – review & editing, Validation, Supervision, Software, Methodology, Conceptualization.

## Financial support

The creation and evaluation of the e-learning program were supported by Covea and GMF mutual insurance companies. The scientific study evaluating the e-learning program and the Tipi® emotional regulation method was carried out by Montpellier University Hospital.

## Declaration of competing interest

The authors declare that they have no known competing financial interests or personal relationships that could have appeared to influence the work reported in this paper.

## Data Availability

The authors do not have permission to share data.

## Appendix A Supplementary data

Supplementary materials: Evolution of stress and emotional states following Tipi® emotional regulation in French teachers and hospital workers (2020–2021) Image 1
