# Supplementary material for: Evaluation of a large-scale E-learning program on emotional regulation Tipi® for hospital workers and teachers in France during the COVID−19 pandemic
Source: Prev Med Rep. 2025 Aug 16;58:103208. doi: 10.1016/j.pmedr.2025.103208 (PMC12491729; doi:10.1016/j.pmedr.2025.103208)
Supplement: Supplementary file 1 — Supplementary materials: Evolution of stress and emotional states following Tipi® emotional regulation in French teachers and hospital workers (2020–2021) [file mmc1.docx]

**Supplementary table 1:** Median levels of stress and emotions (fear, anxiety, sadness, anger) at baseline, one and three months, and their absolute variation in the total French study population and in the teacher and hospital worker subgroups enrolled in the Tipi^®^ emotional regulation method (France, 2020-2021).

|  |  | Baseline | One month | Three months | One month - Baseline | P² | Three months - Baseline | P² |
| --- | --- | --- | --- | --- | --- | --- | --- | --- |
| Stress | Total population | 6 (5;8) | 5 (3 ;6) | 5 (3 ;7) | -1 (-2;1) | <0.01 | -1 (-2;0.5) | <0.01 |
|  | Teachers | 7 (5;8) | 5 (3 ; 6.5) | 5 (4 ;7) | -1(-2;0) | <0.01 | -1(-2.5;1) | <0.01 |
|  | Hospital workers | 6 (5:8) | 5 (3 ;6) | 5 (3 ;7) | -1(-2;1) | <0.01 | -1(-2;0) | <0.01 |
|  | *P (teachers vs. hospital workers) ^1^* | *0.7* | *0.8* | *0.2* |  |  |  |  |
| Fear | Total population | 5(3;7) | 4(2 ;6) | 4(2 ;6) | -1(-2;1) | <0.01 | 0(-3;1) | <0.01 |
|  | Teachers | 5(2;7) | 5(2;6) | 5(2;6) | -1(-2;1) | 0.06 | 0(-2;1) | 0.2 |
|  | Hospital workers | 5 (3;7) | 4 (2 ;6) | 3 (1 ;5) | -1(-2;1) | <0.01 | -1(-3;0) | <0.01 |
|  | *P (teachers vs. hospital workers) ^1^* | *0.5* | *0.9* | *0.03* |  |  |  |  |
| Anxiety | Total population | 7 (5;8) | 5 (3 ;7) | 5 (3 ;7) | -1(-2;1) | <0.01 | -1(-3;0) | <0.01 |
|  | Teachers | 7 (5;8) | 5 (3 ;6) | 6 (4 ;7) | -1(-2.5;0) | <0.01 | -1(-3;0) | <0.01 |
|  | Hospital workers | 6 (4;8) | 5 (3 ;7) | 6 (4;7) | -1(-2;1) | <0.01 | -1(-3;0) | <0.01 |
|  | *p (teachers vs. hospital workers) ^1^* | *0.1* | *0.7* | *< 0.01* |  |  |  |  |
| Sadness | Total population | 5(2;7) | 3(1 ;6) | 3(2;5) | -1(-2;1) | <0.01 | -1(-2;1) | <0.01 |
|  | Teachers | 5(2;7) | 4(2;6) | 4(2;6) | -1(-2;1) | <0.01 | -1(-2;1) | 0.2 |
|  | Hospital workers | 4(2;7) | 3(1 ;6) | 2(1 ;5) | -1(-2;1) | <0.01 | -1(-2;1) | <0.01 |
|  | *p (teachers vs. hospital workers) ^1^* | *0.7* | *0.08* | *0.05* |  |  |  |  |
| Anger | Total population | 5(2;7) | 3(2;6) | 4(2;6) | -1(-3;1) | <0.01 | -1(-2;1) | <0.01 |
|  | Teachers | 5(2;7) | 4(2;5) | 4(2;6) | -1(-3;0.5) | <0.01 | 0(-2;1) | 0.3 |
|  | Hospital workers | 5(2;7) | 3(1 ;6) | 3(2;6) | -1(-3;1) | <0.01 | -1(-3;1) | <0.01 |
|  | *p (teachers vs. hospital workers) ^1^* | *0.4* | *0.8* | *0.1* |  |  |  |  |

Scores range from zero (none) to 10 (maximum value) and are expressed with median (interquartile range).

^1^ Wilcoxon Mann-Whitney test

² Paired Wilcoxon test or paired Student’s t-test depending on the normality.

**Supplementary table 2:** Relative variation (%) in VAS scores for stress and emotions between baseline and one month and between baseline and three months, in the total French study population and in the teacher and hospital worker subgroups, enrolled in the Tipi^®^ emotional regulation method (France, 2020-2021).

|  |  | Total population | | | Teachers | | | | Hospital workers | | |
| --- | --- | --- | --- | --- | --- | --- | --- | --- | --- | --- | --- |
|  | Variation from baseline | N | Median (IQR) | p^1^ | | N | Median (IQR) | p^1^ | N | Median (IQR) | p^1^ |
| Stress | One month | 212 | -14 (-38 ; 13) | <0.01 | | 84 | -13 (-33;0) | 0.07 | 128 | -14 (-38;17) | 0.01 |
|  | Three months | 152 | -14 (-39 ; 6) | <0.01 | | 64 | -13 (-33;15) | 0.2 | 88 | -17 (-40;0) | <0.01 |
| Fear | One month | 215 | -14 (-40 ; 33) | 0.8 | | 84 | -13 (-40;33) | 1.0 | 131 | -17 (-40;40) | 0.7 |
|  | Three months | 155 | 0 (-50 ; 33) | 0.5 | | 65 | 0 (-43;33) | 0.9 | 90 | -6 (-56;33) | 0.5 |
| Anxiety | One month | 215 | -14 (-38 ; 11) | <0.01 | | 84 | -15 (-39;0) | 0.03 | 131 | -14 (-38;14) | 0.07 |
|  | Three months | 155 | -17 (-50 ; 0) | <0.01 | | 65 | -13 (-38;0) | 0.06 | 90 | -24 (-50;0) | <0.01 |
| Sadness | One month | 215 | -17 (-50 ; 17) | 0.04 | | 84 | -15 (-43;15) | 0.2 | 131 | -20 (-50;17) | 0.1 |
|  | Three months | 155 | -25 (-50 ;40) | 0.4 | | 65 | -22 (-50;50) | 1.0 | 90 | -25 (-60;14) | 0.3 |
| Anger | One month | 215 | -13 (-50 ;13) | <0.01 | | 84 | -21 (-50;6) | 0.01 | 131 | -13 (-50;13) | 0.06 |
|  | Three months | 155 | -11 (-50 ; 40) | 0.9 | | 65 | 0 (-38;40) | 0.9 | 90 | -11 (-50;40) | 0.8 |

IQR: interquartile range

Scores range from zero (none) to 10 (maximum value)

Relative variations in visual analogue scale score are calculated as a percentage of the score at baseline and are expressed with the median (interquartile range).

^1^Paired Wilcoxon test.

**Supplementary figure 1:** Median stress and emotion levels (fear, anxiety, sadness, anger) at baseline, one and three months according to sex, in the teacher and hospital worker subgroups enrolled in the Tipi^®^ emotional regulation method (France, 2020-2021).

Scores range from zero (none) to 10 (maximum value)

Bars represent the interquartile range. ***** indicates a significant difference between groups at the indicated timepoint with p<0.05 (Wilcoxon Mann-Whitney test).


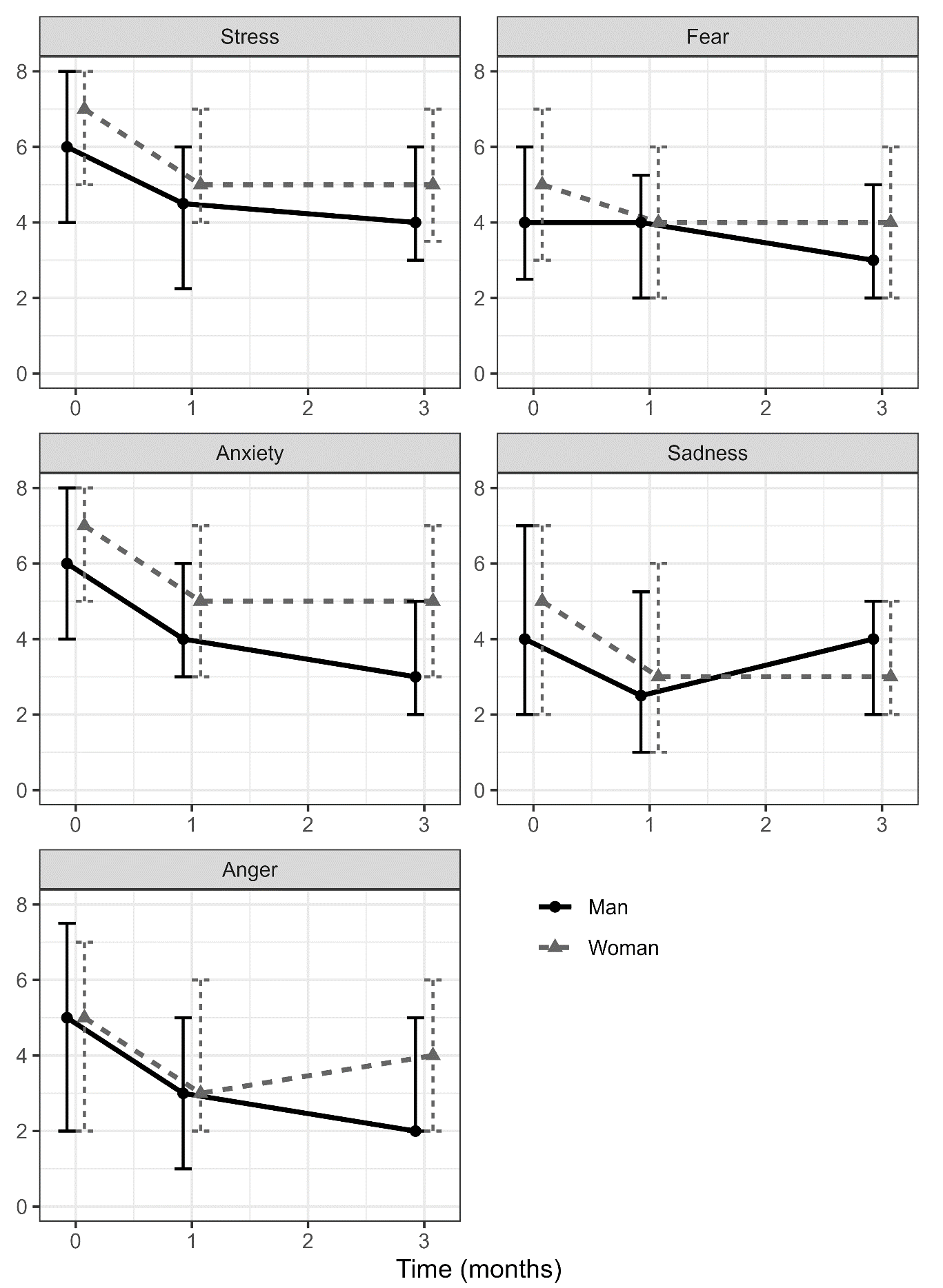


*****

*****

**Supplementary figure 2:** Median stress and emotion levels (fear, anxiety, sadness, anger) at baseline, one and three months according to age group, in the French teacher and hospital workers subgroups, enrolled in the Tipi^®^ emotional regulation method (France, 2020-2021)

Scores range from zero (none) to 10 (maximum value)

Bars represent the interquartile range. No significant difference between groups was noted (Kruskal- Wallis test).
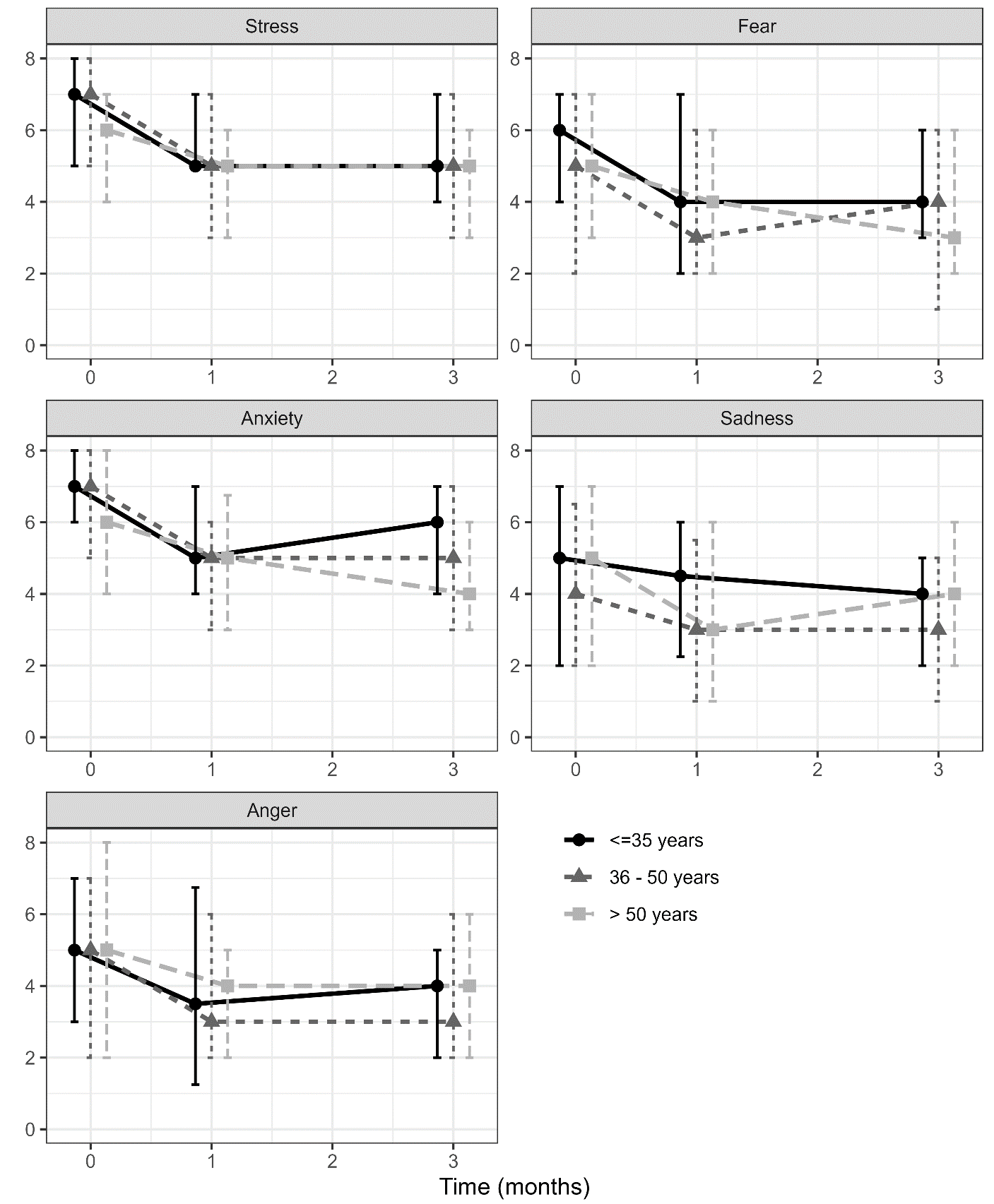


**Supplementary figure 3:** Median stress and emotion levels (fear, anxiety, sadness, anger) at baseline, one and three months according to occupation among the French teacher subgroup, enrolled in the Tipi^®^ emotional regulation method (France, 2020-2021)

Scores range from zero (none) to 10 (maximum value)

Bars represent the interquartile range. No significant difference between groups was noted (ANOVA or Kruskal- Wallis test).
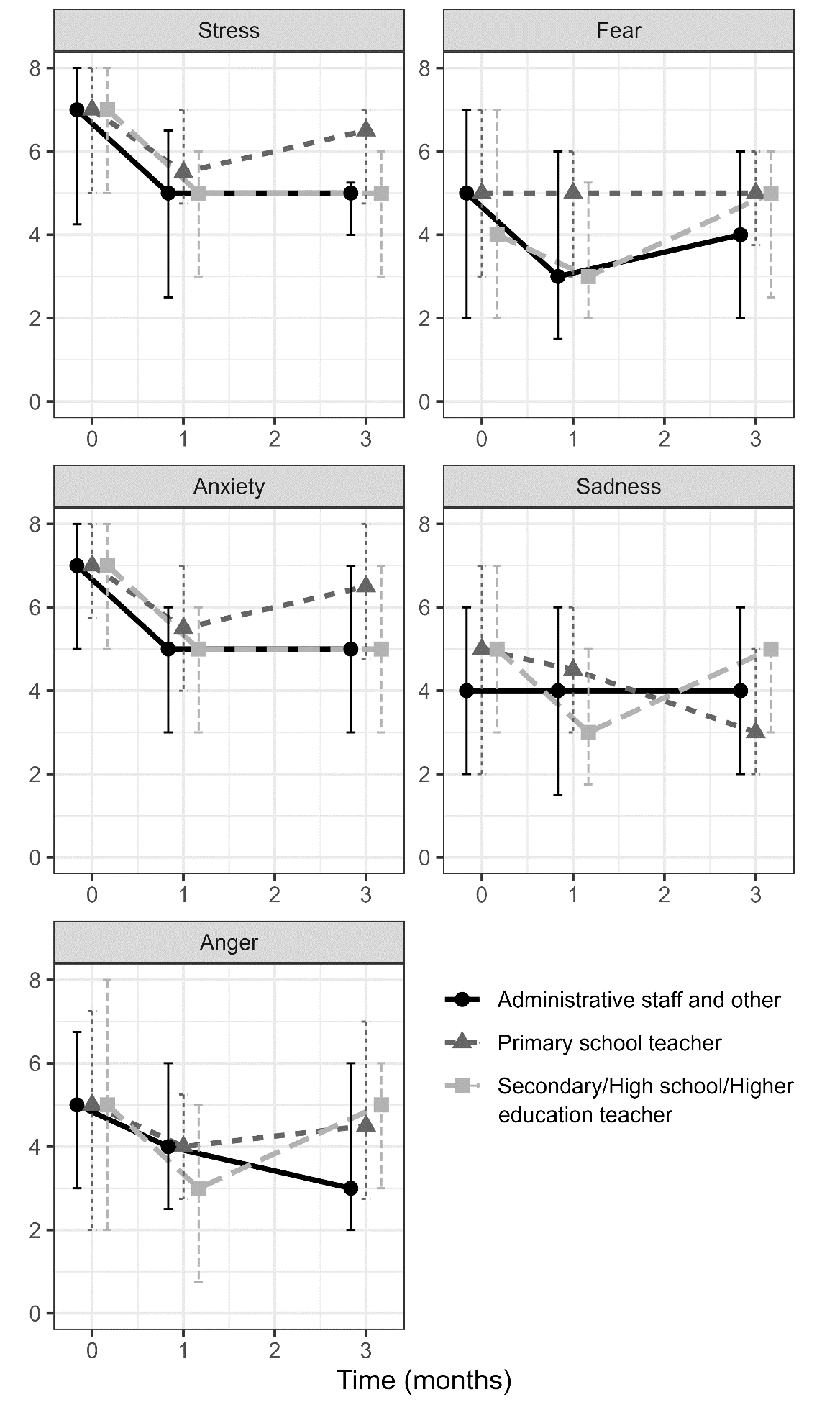


**Supplementary figure 4:** Median stress and emotion levels (fear, anxiety, sadness, anger) at baseline, one and three months according to occupation among the French hospital worker subgroup, enrolled in the Tipi^®^ emotional regulation method (France, 2020-2021)

Scores range from zero (none) to 10 (maximum value)

Bars represent the interquartile range. ** indicates a significant difference between groups at the indicated timepoint with p<0.01 (Kruskal-Wallis test).


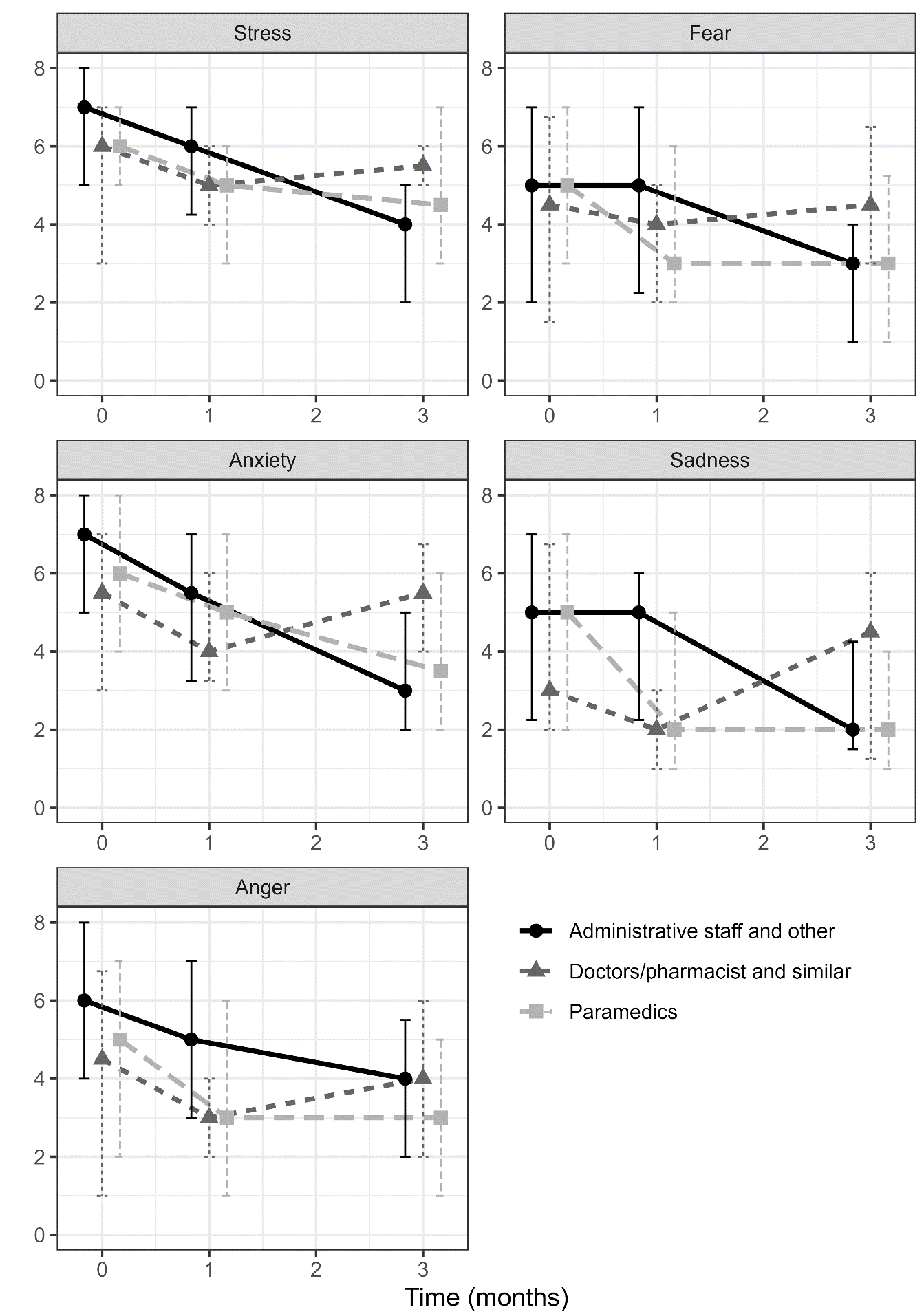


******

******
